# Supplementary material for: kHz-precision wavemeter based on reconfigurable microsoliton
Source: Nat Commun. 2023 Jan 12;14:169. doi: 10.1038/s41467-022-35728-x (PMC9834224; doi:10.1038/s41467-022-35728-x)
Supplement: Supplementary file 1 — Supplementary Information [file 41467_2022_35728_MOESM1_ESM.pdf]

Supplementary Information for "kHz-precision wavemeter based on reconfigurable microsoliton"

Rui Niu,[1,2,*] Ming Li,[1,2,*] Shuai Wan,[1,2] Yu Robert Sun,[2,3]
Shui-Ming Hu,[2,3,4] Chang-Ling Zou,[1,2,†] Guang-Can Guo,[1,2] Chun-Hua Dong,[1,2,†]
[1]CAS Key Laboratory of Quantum Information,
University of Science and Technology of China, Hefei 230026, China.
[2]CAS Center For Excellence in Quantum Information and Quantum Physics,
University of Science and Technology of China, Hefei 230026, China.
[3]Department of Chemical Physics,
University of Science and Technology of China, Hefei 230026, China.
[4]Institute of Advanced Science Facilities, Shenzhen, 518107, China,
[*]These two authors contributed equally to this work.
[†]Corresponding author. E-mail: clzou321@ustc.edu.cn; chunhua@ustc.edu.cn;

(Dated: December 8, 2022)

SUPPLEMENTARY NOTE 1 - THEORETICAL MODEL

A. Global tuning of the cavity dispersion

The resonant frequencies ω_j of a family of modes are determined by the refractive index $n(\omega,\overrightarrow{r})$ and the geometry of the cavity following the Helmholtz equation

$$\left[\nabla^2+n^2(\omega,\overrightarrow{r})\frac{\omega^2}{c^2}\right]\psi(\omega,\overrightarrow{r})=0, \tag{1}$$

where $n(\omega,\overrightarrow{r})$ is the distribution of refractive index at frequency ω, $\psi(\omega,\overrightarrow{r})$ is the distribution of the electric field of the resonant mode. The material dispersion and geometry of the cavity are included in $n(\omega,\overrightarrow{r})$. This equation gives a group of eigenmodes with a corresponding effective mode index $n_{\mathrm{eff}}(\omega)$, by which the cavity resonance is calculated by $2\pi R n_{\mathrm{eff}}(\omega)=m\frac{2\pi c}{\omega_m}$. Due to the material and geometric dispersion, $n_{\mathrm{eff}}(\omega)$ is frequency dependent, thus the cavity resonances ω_j are not equally spaced and can be expanded in the Taylor series near a mode ω_0

$$\omega_j=\omega_0+jD_1+\frac{j^2}{2}D_2+.... \tag{2}$$

In soliton frequency comb generation, the central frequency of the comb is the pump laser frequency ω_p, which should be nearly resonant with the resonance of the pump mode ω_0, The FSR D_1 is the repetition rate or frequency space of the comb lines. D_2 and the pump detuning $\omega_p-\omega_0$ determines the comb bandwidth. The frequency of the pumped mode is related to $n_{\mathrm{eff}}(\omega)$ by

$$\omega_0\propto\frac{c}{R}\frac{q}{n_{\mathrm{eff}}(\omega_0)}, \tag{3}$$

with q being the absolute mode index of the pumped mode. Near the pump, we have the dispersion

$$D_1\propto\frac{c}{R}\left[\frac{1}{n_{\mathrm{eff}}(\omega_0)}+\frac{\Delta\omega}{n_{\mathrm{eff}}(\omega_0)}\frac{\partial n_{\mathrm{eff}}(\omega)}{\partial\omega}|_{\omega_0}\right], \tag{4}$$

which is also relevant to the dispersion of n_{eff}.

To fully stabilize the soliton state, the comb frequency should be locked to two stable references. For example: (i) locking a single comb line, for example, the pump laser, to a stable optical frequency; (ii) locking the repetition rate D_1 to a stable radio frequency, both of which requires the tunability of these frequencies. However, it remains a great challenge to independently control these two frequencies. Without external injections, the frequency tuning of D_1 is realized by tuning $n_{\mathrm{eff}}(\omega_0)$ via elctro-optic or thermal-optic effects. As inferred from Eqs.(3) and (4), the pumped mode frequency ω_0 is approximately q times more sensitive to n_{eff} than that of D_1, which is usually above 10^3 in a typical microcavity. Consequently, a small change of D_1 will also induce a large drift of ω_0. As a result, the pump laser becomes far-off resonance with the cavity mode and goes out of the soliton step.

This problem can be overcame by tuning the dispersion of n_{eff} in the second term of Eq.(4) while remaining $n_{\mathrm{eff}}(\omega_0)$ unchanged. An effective approach is to adjust the distribution of $n(\omega,\overrightarrow{r})$. For example, if the cavity is filled with two kinds of materials with refractive index of $n_1(\omega_0)$ and $n_2(\omega_0)$, we can change the length L_1 and L_2 of the materials and ensure $n_1(\omega_0)L_1+n_2(\omega_0)L_2$ unchanged so that the resonant frequency of the pump mode does not shift. Since the dispersion of $n_{1,2}(\omega)$ are different, now the dispersion of $n_1(\omega_0)L_1+n_2(\omega_0)L_2$ is changed, so as the frequency of other modes. In this way, we can realize the independent control of the resonant frequency and the FSR of the cavity.

B. Two-temperature thermal tuning

1. *Self stabilization of single resonance*

To realize the tuning of D_1 while leaving ω_{p} unchanged, we introduce two thermal tuning mechanisms into the system by pumping two different spatial modes by two lasers and use the two-temperature model (TTM) to investigate the performance. To stabilize the cavity resonance of the pumped mode, the effective cross-phase interaction between two different mode families is introduced by the thermal effect. Under coherent driven by lasers on modes a and b, the dynamics of the system follows

$$\frac{d}{dt}a=(-i\Delta_a-\kappa_a)a+\varepsilon_a, \tag{5}$$

$$\frac{d}{dt}b=(-i\Delta_b-\kappa_b)b+\varepsilon_b, \tag{6}$$

where ε_j is the driving strength on mode j, the detunings Δ_j depends on the detuning $\delta_j = \omega_{j,0} - \omega_{p,j}$ between the cold cavity resonance $\omega_{j,0}$ and the frequency of the pump laser $\omega_{p,j}$, and also the temperature difference T_j between the laser-heated modes and the environments by

$$\Delta_a = k_{aa}T_a + k_{ab}T_b + \delta_a, \tag{7}$$

$$\Delta_b = k_{bb}T_b + k_{ba}T_a + \delta_b. \tag{8}$$

Since the two modes have different field distributions, their thermal field distributions are different and thus influences differently on the cavity resonances by the coefficients $k_{aa}, k_{ab}, k_{ba,} k_{bb}$. For relatively fast thermal dynamics, the temperatures T_j of the two modes are

$$T_a = \alpha_a |a|^2, \tag{9}$$

$$T_b = \alpha_b |b|^2, \tag{10}$$

where α_j depends on the material, geometry, heat capacity of the cavity as well as the mode distribution. At steady state $\frac{d}{dt}a = \frac{d}{dt}b = 0$, we arrive at

$$T_a \left[\kappa_a^2 + (k_{aa}T_a + k_{ab}T_b + \delta_a)^2\right] = \alpha_a \varepsilon_a^2, \tag{11}$$

$$T_b \left[\kappa_b^2 + (k_{bb}T_b + k_{ba}T_a + \delta_b)^2\right] = \alpha_b \varepsilon_b^2. \tag{12}$$

For a small change $d\delta_b$ of the b mode (other parameters of the system are fixed), the corresponding changes dT_a, dT_b of the two temperatures fulfill

$$f_{aa}dT_a + f_{ab}dT_b = 0 \tag{13}$$

$$f_{ba}dT_a + f_{bb}dT_b = -f_{bx}d\delta_b, \tag{14}$$

where f_{aa} and f_{ab} are the derivative of T_a and T_b with respect to the left side of Eq. 11, f_{ba}, f_{bb} and f_{bx} are the derivative of T_a, T_b δ_b with respect to the left side of Eq. 12. Then we can solve these two equations and obtain the temperature changes dT_a and dT_b. The change of the resonance frequency of mode a due to the change of the driving frequency of mode b is derived as

$$\begin{aligned}\delta\omega_a &= k_{aa}dT_a + k_{ab}dT_b \\ &= \frac{2k_{ab}dT_b\left(\Delta_a^2 + \kappa_a^2\right)\Delta_b}{4k_{ab}k_{ba}dT_a dT_b - \left(2k_{aa}dT_a\Delta_a + \Delta_a^2 + \kappa_a^2\right)\left(2k_{bb}dT_b\Delta_b + \Delta_b^2 + \kappa_b^2\right)} d\delta_b.\end{aligned} \tag{15}$$

By controlling the frequency of and amplitude of the drive laser on mode b, the system can be tuned to the special biased point $\Delta_b = k_{bb}T_b + k_{ba}T_a + \delta_b \approx 0$. Near this point, the resonant frequency of mode a remains nearly unchanged even though the frequency of the drive laser on mode b is shifted.

2. *Independent tuning of FSR*

In addition to the two pumped modes, the resonant frequencies of other modes belonging to the same mode family with a are also influenced by the thermal effect. Similarly, we derive the thermal-optic shift of an arbitrary mode

$$\delta\omega_j = \widetilde{k}_{aa}dT_a + \widetilde{k}_{ab}dT_b. \tag{16}$$

It should be noted that, due to the geometry dispersion, optical mode of different wavelengths responses different to the same thermal field, thus $\widetilde{k}_{aa} \neq k_{aa}$ and $\widetilde{k}_{ab} \neq k_{ab}$. As a result, the mode adjacent to the pumped mode has different frequency shift and the FSR of the soliton mode family is changed.

Under the self stabilization condition, the resonant frequency of mode a remains nearly unchanged when the frequency of the drive laser on mode b is shifted. Thus the change of resonant frequency of mode a and mode b fulfill

$$k_{aa}dT_a + k_{ab}dT_b = 0, \tag{17}$$

$$k_{bb}dT_b + k_{ba}dT_a = \Delta\omega_b. \tag{18}$$

The thermal-optic shift of mode j is derived as

$$\delta\omega_j = -\frac{\widetilde{k}_{aa}k_{ab}\Delta\omega_b}{k_{aa}k_{bb} - k_{ab}k_{ba}} + \frac{\widetilde{k}_{ab}k_{aa}\Delta\omega_b}{k_{aa}k_{bb} - k_{ab}k_{ba}}. \tag{19}$$

The value of $k_{aa}, k_{ab}, k_{ba}, k_{bb}, \widetilde{k}_{aa}, \widetilde{k}_{ab}$ at different wavelengths is obtained by using the software Comsol Multiphysics to calculate the slope of the resonance mode response to various temperature fields. Here, we choose the simulation process of k_{aa} for example, by applying temperature field T_a of varying amplitude, we calculate the corresponding resonance frequency of mode a. The value of k_{aa} is calculated from the slope of resonance frequency and the amplitude of temperature field. The frequency shift of the auxiliary laser is obtained from the experimental results. In our experiment, the pump mode for soliton generation is chosen as the fundamental mode while the auxiliary mode is set as the higher order mode, as shown in Supplementary Fig. 1(D). The corresponding resonance shift of the pump mode with varying auxiliary laser frequency is shown in Supplementary Fig. 1. By applying such a TTM model, we realize the real-time control FSR of the microcavity while leaving individual mode resonance unchanged.

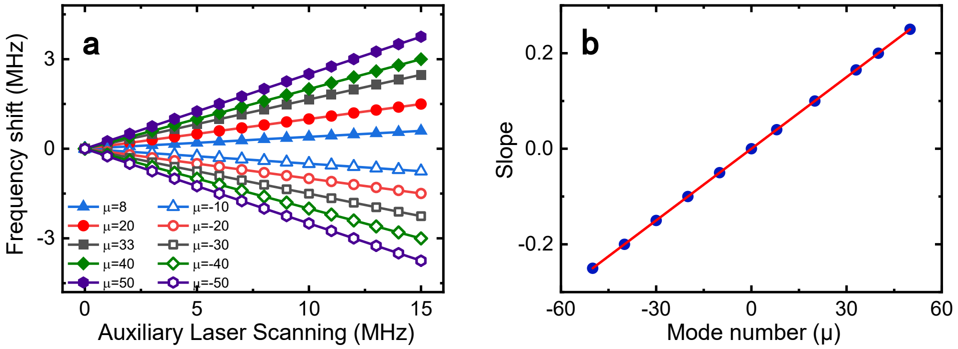

Supplementary Figure 1. **a**, The calculated frequency shift of the optical modes with different mode number μ by changing the auxiliary laser frequencies through the two-temperature model. **b**, the calculated slopes of the simulated frequency shifts for different μ.

SUPPLEMENTARY NOTE 2 - EXPERIMENTAL SETUP AND SAMPLE CHARACTERIZATION

A detailed diagram of the wavemeter setup is provided in Supplementary Fig. 2. The pump laser around 1550 nm is coupled into the microrod cavity for the soliton generation, meanwhile the auxiliary laser is coupled into the microcavity from the opposite direction for thermal balance. Because the Q factor of the pump mode is about twice of the Q factor of the auxiliary mode, the power of the pump laser and the auxiliary laser are amplified by the erbium doped fiber amplifier (EDFA) for launched power about 100 mW and 380 mW, respectively. The transmissions of the pump laser and the auxiliary laser are monitored on the oscilloscope. The frequency of the pump laser is locked to the reference cavity with a finesse of 250 through PDH locking. After generation of the soliton microcomb, the repetition rate of the soliton microcomb is down converted to sub-GHz through the electro optic modulator (EOM) and locked to the local oscillator (LO) through phase locking, where the feedback signal is connected to the auxiliary laser. And the frequency of the repetition rate could be tuned during the sweep of the local oscillator frequency.

The microrod for generating the soliton microcomb is fabricated by heating with the CO_2 laser beam. The detail of the fabrication process of the microcavity can be found in the previous work [1]. The photo of the microrod is shown in Supplementary Fig. 3(a), with a diameter of 1.07 mm. The quality factor of the optical modes used for the soliton generation is 4.76×10^7, as shown by the transmission spectrum in Supplementary Fig. 3(b). After employing the auxiliary laser at the appropriate detuning, clear soliton steps emerge on the pump transmission, as shown in the blue curve of Supplementary Fig. 3(c). By scanning the pump laser from the blue detuning to the red detuning, we reached the multi-soliton state (I), two-soliton state (II) and single soliton state (III), and the corresponding soliton spectra are shown in the Supplementary Figs. 3(d)-(f).

In the experiment, we use a weak signal laser (about 50 μw) to detect the frequency changes of comb lines and the corresponding optical modes for different detunings (Δ) of the auxiliary laser when the single soliton is maintained and the pump laser is fixed. The detailed setup is shown in Supplementary Fig. 4(a). The signal laser is launched into the microcavity from the opposite direction of the pump laser to avoid the distribution of the soliton generation. The signal laser and the auxiliary laser are separated by the dense wavelength division multiplexing (DWDM). The signal laser is scanned around the comb lines to detect the frequency change. The typical transmission spectra of the signal laser ($\mu = 0$, $\mu = -10$, $\mu = 33$) with different auxiliary laser detunings are shown in the Supplementary Fig. 4(b). The small peak in the transmission spectra is the beating signal between the comb line and the signal laser, which is filtered with the low bandwidth detector

The evolution of the repetition rate of the soliton is chracterized by the real-time spectrum analyzer under different locking conditions. Figure 5 shows the measured linewidth and evolution of the repetition rate for the condition only the pump laser is locked and the condition both the pump laser and repetition rate are locked. When the pump laser frequency is stabilized to the

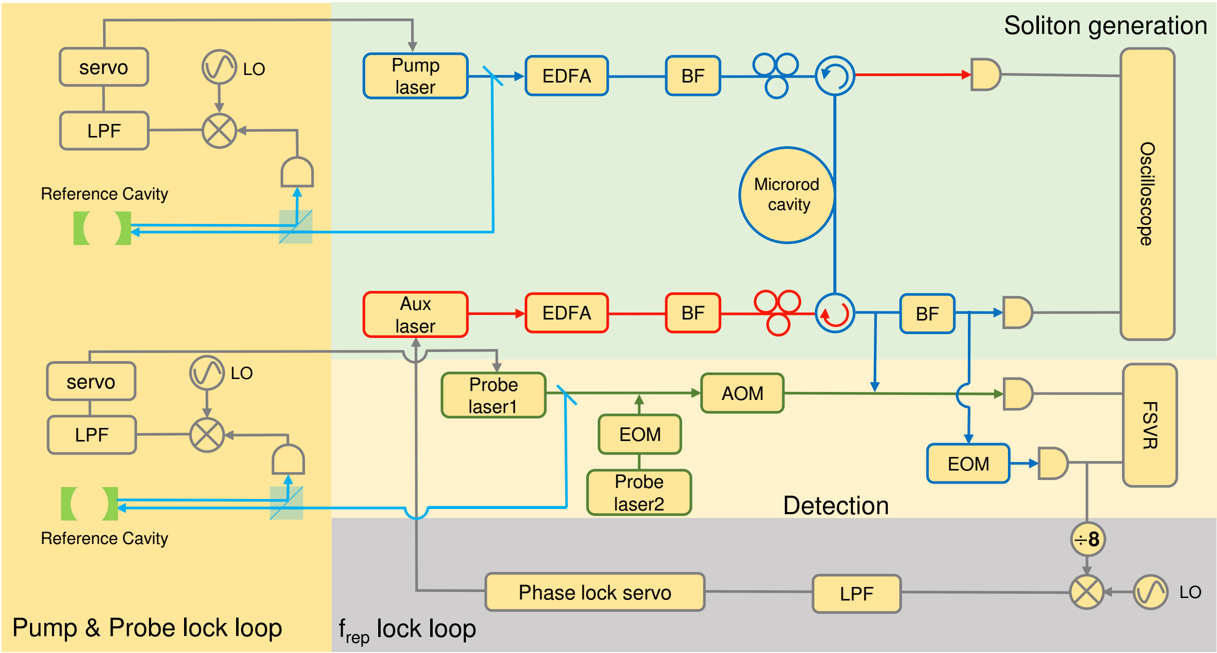

Supplementary Figure 2. **Experimental setup.** EDFA: erbium doped fiber amplifier; BF: band pass filter; AOM: acousto-optic modulator; EOM: electro-optic modulator; LO: local oscillator; LPF: low-pass filter; FSVR: real-time spectrum analyzer.

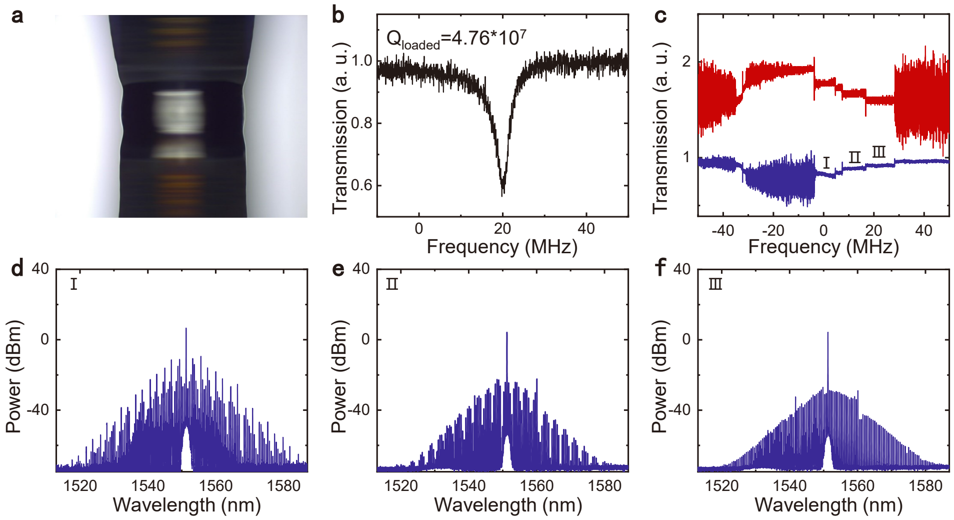

Supplementary Figure 3. **Sample characterization and soliton generation.** **a**, The photo of the microrod cavity with a diameter of about 1.07 mm. **b**, The typical transmission spectrum of a mode around 1550 nm with the quality factor of 4×10^7. **c**, Transmission of the pump laser (blue curve) and the auxiliary laser (red curve) when the pump laser sweep across the resonance at 47.1 kHz/μs. **d-f**, Optical spectra of the multi-soliton state (I), two-soliton state (II) and the single soliton state (III).

reference cavity, the linewidth of the repetition is suppressed to 11.3 kHz, as shown in the Supplementary Fig. 5(a). However, the long term stability of the repetition rate is still constrained by the stability of the auxiliary laser. As shown in the Supplementary Fig. 5(c), during the 3 seconds measurement period, the fluctuation of the repetition rate exceeds 5 kHz. Furthermore, by locking the repetition rate (auxiliary laser) to the local oscillator, the linewidth of the repetition rate is compressed to 9.2 kHz, and the long term stability is significantly improved, as shown in Supplementary Figs. 5(b) and (d).

We also character the frequency stability of the unlocked and locked pump laser, by introducing an reference laser around 1542 nm (stabiλlaser 1542, linewidth $\sim$ 300Hz at short term, frequency stability of 3×10^{-13} at 1 s measurement time). As shown in Supplementary Fig. 6, comparing to the unlocked state (black line), the frequency stability of locked state (red line) has a significant improvement (11.8 kHz at 1 s measurement time and 576 Hz at 512 s measurement time). The pump laser shows a higher frequency stability compare to the comb line, which could be explained as part of the frequency drift of comb line is

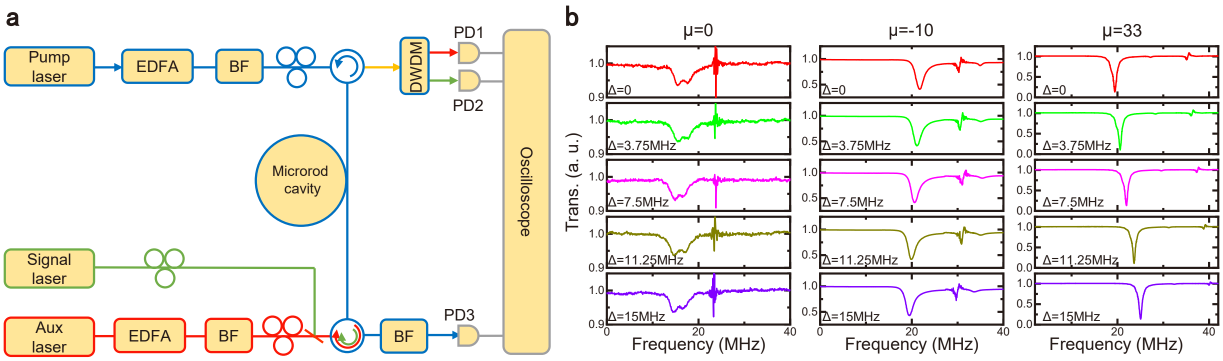

Supplementary Figure 4. **a**, EDFA: erbium doped fiber amplifier; BF: band pass filter; DWDM: dense wavelength division multiplexing; PD: photon detector. **b**, Typical transmission spectra of signal laser with different auxiliary frequency.

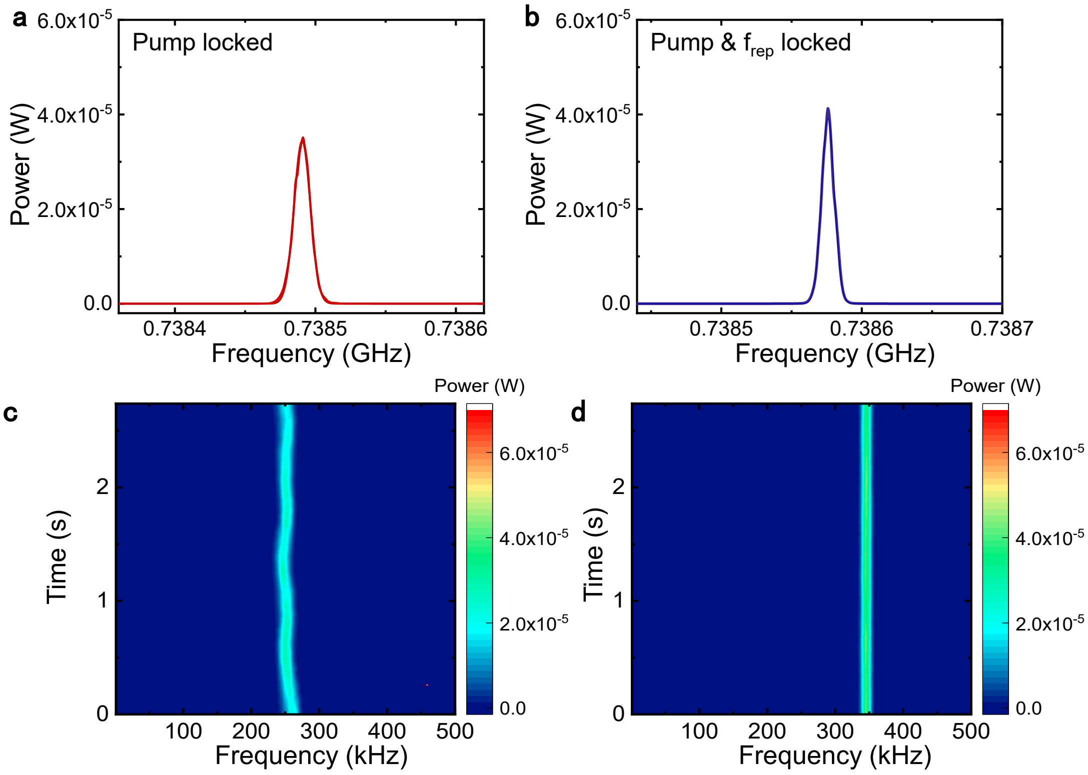

Supplementary Figure 5. **Repetition rate linewidth and evolution.** **a**, The linewidth of the repetition rate (measurement time of 10ms) when the pump laser is locked to a reference cavity. **b**, The linewidth of the repetition rate when the pump laser is locked to the reference cavity and the repetition rate is locked to the local oscillator. **c**, The evolution of the repetition in 3s measurement period when the pump laser is locked. (d) The evolution of the repetition in 3s measurement period when the pump laser and the auxiliary laser are both locked.

contributed by the uncertainty of repetition rate.

SUPPLEMENTARY NOTE 3 - WAVEMETER PERFORMANCE

To demonstrate the high-precision of the wavemeter, three under-test probe laser beams are used in the experiment. One probe laser with wavelength around 1555 nm is locked to the reference cavity with a finesse of 250, another two probe laser beams are sidebands of phase modulated acetylene-stabilized fiber laser (stabiλlaser 1542). The probe laser beams are launched together and mixed with the frequency stabilized soliton microcomb for wavelength measurement. The generated beat note signals and the corresponding repetition rates are recorded on the real-time spectrum analyzer. The wavelength of probe lasers can be determined unambiguously by scanning the frequency of repetition rate and switching the AOM frequency. Furthermore,

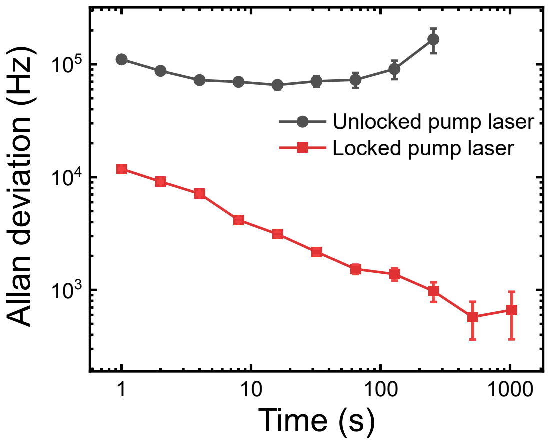

Supplementary Figure 6. The Allan deviation of unlocked and locked pump laser. Allan deviation of the pump laser for unlocked state (black line) and locked state (red line). At measurement time of 1 s, the frequency stability of the locked pump laser reaches 11.8 kHz. Error bars represent a 68% confidence interval.

we also measured the corresponding low RF noise spectra of comb lines with tuning the repetition rate. In the experiment, we used a notch filter to filter the pump laser. As shown in Supplementary Fig. 7, in the whole process, the repetition remains a narrow linewidth and the RF spectrum remains very low noise in such range, which indicates the comb lines remain coherent while they are being tuned.

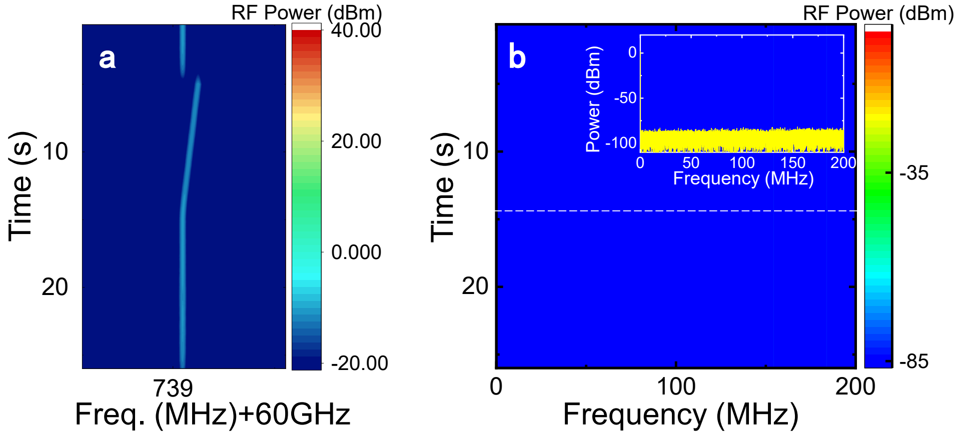

Supplementary Figure 7. The real-time evolution of the repetition rate **a** and the corresponding low RF noise spectra **b** of the soliton state while tuning the repetition rate. Inset: the spectrum corresponding to the dot line in **b**.

The frequency of probe laser is expressed as $\omega_{\mathrm{j}} = f_{\mathrm{p}} + \mu f_{\mathrm{rep}} \pm \Omega_{\mathrm{j}}$. We have to determine the order of μ and the sign of Ω_j to confirm the frequency of the target probe lasers, where f_p can be calibrated with a frequency standard.

The sign of μ determines whether the probe laser locates at the blue or red side of the pump laser, and the sign before Ω_j determines whether the probe laser locates at the blue or red side of the adjacent comb line. The absolute value of μ can be determined by sweeping the frequency of the repetition rate, as shown in Supplementary Fig. 8. Taking Supplementary Fig. 8(a) for example, the repetition rate is tuned from 60.739059 GHz to 60.739143 GHz within 10 s, at the same time Ω_{j} is scanned from 3.634282 GHz to 3.635795 GHz. The ratio of the slopes of repetition rate and the beating note represents the absolute value of the beating comb line. Here the absolute value is $|\mu| = 18.01$.

Furthermore, without the sign of the ratio of the slopes, there are also ambiguity information of the position of the probe laser. For example, if the frequency of the beat note signal increases with the repetition rate, the probe laser locals at the blue detuning of the pump laser and the red detuning of the comb line or at the red detuning of the pump laser and the blue detuning of the comb line. Thus, to determine the sign of $|\mu|$ and Ω_j, we introduce an AOM and change the modulation frequency from 80 MHz to 75 MHz. If the beating signal increases along with the frequency shift, the probe laser locals at the red detuning of the pump laser and the red detuning of the comb line. Combining with the sweeping of repetition rate and frequency shift of probe laser, the location of probe laser is unambiguously determined. Details of the sign are shown in the following table.

Similar to traditional wavemeter, our system also should be calibrated. Here, we used the acetylene-stabilized fiber laser

Sign of ratio between slope of the repetition rate and frequency shift	The f_{beat} shift vs AOM frequency shift	Sign of μ	Sign of Ω_j
+	+	-	+
+	-	+	-
-	+	+	+
-	-	-	-

Supplementary Table I. Determine μ and the sign of Ω_j from the slope of the repetition rate and frequency shift induced by the AOM.

(stabiλlaser 1542, 194.369489384(5) THz) to calibrate one comb line around 1542nm and deduce that the absolute frequency of pump laser is 193.297542104THz. Then we can get the target frequency of the probe laser to be 194.334206092THz, 194.387130884THz, 192.754730992THz, respectively. Furthermore, for a compact system and calibration-free system, for example, the pump laser or comb line could be referenced to the atomic or molecular transitions [2, 3]. Typical linewidth of the atomic transitions reaches several MHz and the corresponding optical frequency standard accuracy reaches several kHz (1s measurement time).

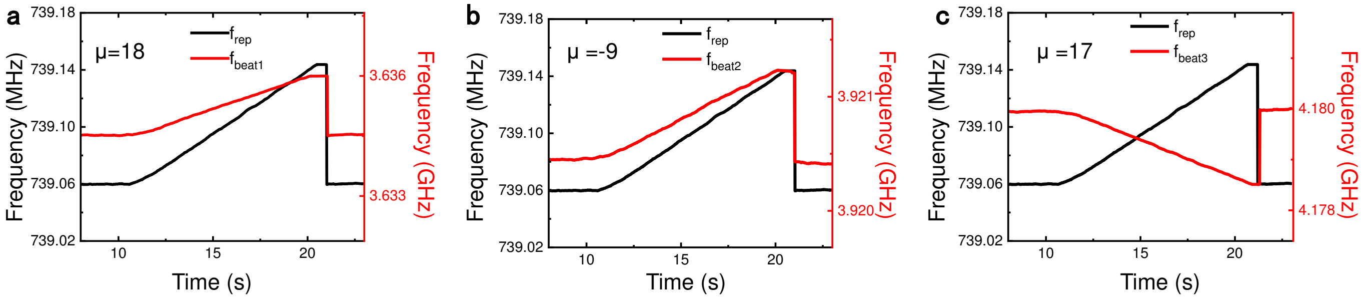

Supplementary Figure 8. Calculation of $|\mu|$ from the real time spectrum, which are obtained from Figs. 2(**c-e**) in the main text.

In addition, we could estimate the locking bandwidth from the repetition rate response in Supplementary Fig. 8. The repetition rate is tuned suddenly and the system response around 1ms, as shown the black curves in Supplementary Fig. 8. It means the locking bandwidth in our system reaches at least 1kHz.

SUPPLEMENTARY REFERENCES

[1] R. Niu, S. Wan, Z.-Y. Wang, J. Li, W.-Q. Wang, W.-F. Zhang, G.-C. Guo, C.-L. Zou, and C.-H. Dong, "Perfect soliton crystals in the high-q microrod resonator," IEEE Photonics Technology Letters **33**, 788 (2021).

[2] Z. L. Newman, V. Maurice, T. Drake, J. R. Stone, T. C. Briles, D. T. Spencer, C. Fredrick, Q. Li, D. Westly, B. R. Ilic, *et al.*, "Architecture for the photonic integration of an optical atomic clock," Optica **6**, 680 (2019).

[3] Roy Zektzer, Matthew T. Hummon, Liron Stern, Yoel Sebbag, Yefim Barash, Noa Mazurski, John Kitching, and Uriel Levy, "A Chip-Scale Optical Frequency Reference for the Telecommunication Band Based on Acetylene," Laser & Photonics Reviews **14**, 1900414 (2020)
